# Aid fragmentation and volatility in the Pacific

**DOI:** 10.1002/app5.321

**Published:** 2021-05-04

**Authors:** Terence Wood, Imogen Nicholls

**Affiliations:** ^1^ Development Policy Centre, Crawford School of Public Policy The Australian National University Canberra ACT Australia; ^2^ The Australian National University Canberra ACT Australia

**Keywords:** aid fragmentation, aid volatility, Australia, China, Pacific

## Abstract

In this article we comprehensively document aid volatility (short‐term changes in aid flows) and aid fragmentation in the Pacific. We study two types of fragmentation: fragmentation across countries and fragmentation across projects. Our research draws on a new dataset compiled by the Lowy Institute. The dataset includes aid flows to the Pacific from non‐traditional donors such as China. This allows us to undertake the first‐ever study of Pacific aid volatility and fragmentation factoring in non‐traditional donors. We contrast the Pacific with other regions, finding that while fragmentation across donors is less in the Pacific, project fragmentation is worse, as is aid volatility. We find fragmentation across donors is increasing in the Pacific. We find a similar trend for fragmentation across projects. We find no evidence that non‐traditional donors such as China are driving these trends. However, we find some evidence that non‐traditional donors give more volatile aid.

## 1 INTRODUCTION

Foreign aid is important in the Pacific: 11 of the world's 20 most aid‐dependent countries are in the region. Even larger Pacific countries, such as Papua New Guinea, are still more aid dependent than the typical developing country globally. 1 Both aid volatility (aid flows that change in volume rapidly year on year) and aid fragmentation (aid being excessively divided across countries or projects) have the potential to reduce the effectiveness of aid. In a region where aid is so important, volatility and fragmentation are in particular need of attention.

In this article we comprehensively document aid volatility and fragmentation in the Pacific. We study volatility by using data from the years 2011 to 2017 and quantify the extent to which aid fluctuates year on year. As we do this, we take care to focus on short‐term volatility rather than changes associated with long‐term trends. When we study fragmentation, we look at two types of fragmentation: country fragmentation, in which donors fragment their aid across numerous recipients or in which recipients receive aid from numerous donors; and project fragmentation, in which aid from individual donors or to individual recipients is fragmented over numerous projects.

In conducting this study, we draw upon two detailed datasets. One of the datasets is conventional: OECD aid data. OECD data are comprehensive, but only for so‐called ‘traditional’ aid donors—countries that are members of the OECD's Development Assistance Committee (DAC), and which have typically given aid for many years. The other dataset—one of aid to the Pacific produced by the Lowy Institute (hereafter referred to as Lowy data)—is new. This is the first time it has been used to study aid fragmentation and volatility. The Lowy dataset has the unique advantage of including so‐called ‘non‐traditional’ aid donors such as China.

Drawing upon the Lowy and OECD data, we cover a suite of research areas, all directly related to aid fragmentation and volatility in the Pacific. First, we use OECD data to compare aid fragmentation and volatility between the Pacific and the rest of the world. We do this to answer the question of whether the Pacific suffers more fragmented and volatile aid than other aid recipient regions.

Second, we focus in on the Pacific and use the Lowy data to look at the following research questions about aid fragmentation and volatility:

- Is aid fragmentation across donors increasing or decreasing in the Pacific?
- Is aid fragmentation across discrete aid projects increasing or decreasing in the Pacific?
- Are trends in fragmentation being driven primarily by traditional DAC donors or by non‐traditional donors such as China?
- Which individual Pacific Island countries suffer the worst from aid that is fragmented across donors?
- Which individual Pacific countries suffer the most fragmentation across projects?
- Which Pacific countries suffer the most volatile aid?
- Is there any relationship between different types of aid fragmentation, and between aid fragmentation and aid volatility at the recipient level?

In the third part of our analysis we shift our focus to the donors that give aid to the Pacific. Looking at donors, we study the following questions:

- Which of the 15 largest donors to the region fragment their aid the most across countries within the Pacific?
- Which of these donors fragment their aid the most across projects?
- Which of these donors give the most volatile aid?
- Is there any relationship between different types of aid fragmentation, and between aid fragmentation and aid volatility at the donor level?
- Do non‐traditional donors tend to, on average, give more fragmented or volatile aid than traditional OECD DAC donors?

Our key findings are that aid volatility is higher in the Pacific than in other regions. Fragmentation across donors is lower. However, once the size of Pacific countries is taken into account, fragmentation across projects is much higher in the Pacific than in other regions. Fragmentation across both donors and projects is increasing in the Pacific, and this increase is not being driven solely by non‐traditional donors—OECD DAC donors are contributing significantly to the increase. When we look at individual Pacific countries, we find a wide range of variation in the extent to which they suffer aid volatility and fragmentation. The issues themselves are not particularly clearly correlated across countries, although in aid recipients project fragmentation is associated with higher aid volatility. The countries that benefit from the lowest donor fragmentation tend to be ones that have particular strategic importance to, or compacts of association with, individual donors. When we study individual donors, we find larger donors fragment their aid more across countries but less across projects. We find that non‐traditional donors are no more guilty of fragmenting aid than traditional donors are. On average, non‐traditional donors are guilty of giving more volatile aid, however.

Our study contributes to the literature by being the first‐ever systematic study of aid fragmentation and volatility in the Pacific. We are also the first study that we are aware of to take advantage of the new Lowy data. These data enable us to examine the impact of non‐OECD donors in the region—a particular advantage as the Pacific is increasingly becoming the focus of geostrategic interest from donors old and new. We further contribute by making our data available online. As a result, other researchers, as well as aid practitioners and Pacific governments, can further study aid fragmentation and volatility in the region.

## 2 LITERATURE

The OECD defines aid fragmentation as ‘aid that comes in too many small slices from too many donors, creating high transaction costs and making it difficult for partner countries to effectively manage’ (Organization for Economic Co‐operation and Development [OECD], 2009, p. 244). It is a widely held view that as aid becomes more fragmented, the quality decreases and it becomes less effective (Kimura et al., 2012; OECD, 2008). There have been a number of international agreements that have set out to reduce aid fragmentation, including the Paris Declaration and the Accra Agenda for Action (OECD, 2008). However, aid fragmentation appears to be growing globally over time (Knack & Rahman, 2007). Aid fragmentation may disproportionately affect countries with small economies and populations by increasing administration costs in countries that can least afford to bear this burden (Knack, 2008).

Aid volatility is typically defined as aid flows that rise or fall substantially in short periods of time (Clarke et al., 2008). The specifics of which types of countries are worst affected by volatility are debated, as are the exact causal mechanisms through which volatility has its effects. However, there is consensus that volatility reduces aid effectiveness. Moreover, as with fragmentation, volatility appears to be becoming worse globally (Birdsall, 2005; Bulíř & Hamann, 2008; Hudson & Mosley, 2008).

Although the level of aid dependence in the Pacific provides good cause to be particularly concerned about fragmentation and volatility, the issues have received surprisingly little study in the region.

As part of what is the most empirically intensive survey of aid flows to the Pacific to date, Dornan and Pryke (2017) discuss aid fragmentation for the Pacific region as a whole, presenting some secondary evidence that aid fragmentation in the Pacific was less than in some other aid recipient regions. Beyond this work, we were unable to find any detailed studies of aid fragmentation to the countries of the Pacific.

Studies of aid volatility in the region are slightly more common, albeit still rare. As part of a detailed piece of econometric analysis focused on small island states more generally, Clarke et al. (2008) cover volatility in the Pacific. Sample size issues prevent them from providing results for the Pacific on its own in their most sophisticated work; however, they were able to provide some separate analysis of the Pacific using simpler methods. For the time period from 1973 to 2003, they show some startling examples of aid volatility experienced by Pacific countries. Dornan and Pryke (2017) also cover aid volatility, showing that aid volatility to the region as a whole was less in the 2000s than in the previous decade, and that volatility tended to be better in the Pacific than in Sub‐Saharan Africa and East Asia. They also found, however, that both trends and levels of volatility varied considerably across the countries of the Pacific. The third existing study of aid volatility, that of Letasi Iulai (2014), is a detailed case study of volatility in the heavily aid‐dependent country of Tuvalu. Iulai finds aid to Tuvalu, and project aid in particular, is highly volatile, a fact that comes at a cost to sound aid management in the country.

## 3 METHODOLOGY

The methods employed in our study differ from existing work in four important ways.

First, except when we compare the Pacific to other regions, we draw on detailed project‐level data from the Lowy Institute's Pacific Aid Map (Dayant & Pryke, 2019). At the time of writing the Pacific Aid Map dataset only contained complete data for 2011 to 2017, but has a major advantage over alternative datasets in that it covers all aid donors to the Pacific. The data are not limited to OECD donors alone, but include non‐traditional donors, most importantly China, which is the third‐largest donor to the Pacific (Dayant & Pryke, 2019). Drawing on this comprehensive new dataset, our study is the first to capture the full extent of aid fragmentation and volatility in the region.

Second, we focus on both donors and recipients, allowing detailed comparisons, not only of which countries suffer fragmentation and volatility the most, but also which donors—and types of donors—contribute to fragmentation and volatility the most.

Third, we look at fragmentation at the country level and at the project level. Existing studies have typically focused on donor fragmentation at the country level, measuring how much individual donors fragment their aid across recipient countries (e.g., Easterly & Pfutze, 2008) or the extent to which recipient countries receive aid that is fragmented across donors (e.g., Dornan & Pryke, 2017). In addition to these approaches we focus on the extent to which aid is fragmented across projects. Our rationale for doing this is that, although country‐level fragmentation is a serious issue that raises both donor and recipient transaction costs, it is not the only type of aid fragmentation that matters. Fragmentation across numerous aid projects will also be inefficient and difficult to manage, even if all projects come from the same donor.

When we calculate country‐level fragmentation we use the Herfindahl–Hirschman Index (HHI), a standard measure in international work (Dornan & Pryke, 2017; Gehring et al., 2017). 2 When we focus on projects we use mean project size (measured as annual project spend in US dollars) when examining donors' project fragmentation, and projects per million people when looking at project fragmentation in aid recipients. These measures of project fragmentation are preferable to alternatives for the following reasons. The HHI, which could technically be used to measure project fragmentation, is bound between zero and one. This is not an issue when dealing with country fragmentation in the Pacific, where fragmentation is relatively low. However, it is an issue when dealing with very highly fragmented phenomena, such as fragmentation across aid projects. This is because the bounded measure fails to capture the full extent of fragmentation in a linear manner once fragmentation is as high as it is when dealing with hundreds or thousands of projects. Mean project size is more appropriate than other alternatives such as simple project counts when focusing on donors, because it takes into account the total volume of aid a donor gives. A donor that gave one billion dollars of aid to the Pacific and spread it across 100 projects could hardly be said to have fragmented their aid to the same degree as a donor that gave $10,000 of aid and also fragmented it across 100 projects. We use projects per million people when studying project fragmentation in recipient countries for a similar reason: a country of 1000 people will suffer much greater drawbacks from aid fragmented across 100 projects than a country of one million people will.

Fourth, because our aim in calculating volatility is to separate short‐term volatility from longer‐term trends, we do not calculate volatility using a simple coefficient of variation calculated as the standard deviation of aid flows over mean aid flows. Rather, we calculate the coefficient of variation of the root mean squared error from an OLS regression in which time is the independent variable and aid volume is the dependent variable. 3 This removes long‐term trends, thereby isolating short‐term volatility, while at the same time normalising the resulting value in the same way conventional coefficient of variation measures do. These attributes make this measure useful for short time series. While some studies use more complex approaches, such as the Hodrick–Prescott filter, to the same effect, we have too few data points to be able to reliably use such approaches (Hudson, 2015).

One aspect of our study of volatility, which is not out of line with some other studies, warrants further explanation. While it is possible to study volatility of aid flows to a recipient country in terms of the volatility of all the aid that country receives from all donors, we have chosen instead to focus on pairs of donors and recipients (what we refer to as donor‐recipient dyads). There is a case for studying volatility of total aid flows to a recipient country, as such volatility may impact on economic features such as aggregate demand and the balance of payments. However, our interest is primarily in aid management, and the largest impact of volatility on both donor and recipient aid management is likely to be felt from rapid changes to aid across donor‐recipient dyads. Reflecting this, when studying volatility in Pacific countries from a recipient perspective, we report on the volatility of the median major donor to the recipient in question. (We focus on major donors because many donors only give small amounts of aid to Pacific countries. Small donors may give volatile aid but, because they give small amounts, volatility is unlikely to cause major practical problems for the recipient.) In practice, focusing on major donors means that, when we calculate median donor volatility for each Pacific country, we take the median across the five largest donors (in terms of aid volume) to the recipient. Other researchers may have other interests, such as studying volatility from all donors, or aggregate volatility at the recipient level. Researchers with such interests can draw upon our online dataset to study these phenomena in the future.

Also, some donors only gave aid to individual Pacific countries in 1 or 2 years out of the 7 years covered in our study. Because it is meaningless to talk of volatility among such occasional aid givers, all of our volatility calculations were undertaken excluding donors that gave aid in less than 3 years across the period of study.

In addition, when calculating volatility and fragmentation, we took the decision to exclude wherever possible aid associated with humanitarian emergencies. Fluctuations in aid spending associated with a cyclone or earthquake are reasonable, and quite different from volatility in aid intended for long‐term development projects. Similarly, it would seem reasonable that more donors might operate in a country in the wake of a disaster. As a result, we excluded humanitarian emergency spending from our calculations of fragmentation wherever possible. (It could well be the case that there can also be too much fragmentation in emergency responses, but studying this specific issue is beyond the scope of our broad overview. Once again, further analysis could be conducted using the data we have placed online.) While we excluded humanitarian spending from all work using the Lowy dataset, and also from OECD data used to study project fragmentation, source data constraints meant we could not exclude humanitarian emergency aid from our regional comparisons of donor fragmentation and volatility.

Finally, there is one limitation with Pacific Aid Map data that could not be overcome in our study and which bears noting. In order to prevent double counting, projects that are funded by multiple donors are counted as multiple projects in the Lowy data. Also, regional projects are broken down across individual recipient countries. These aspects of the source data are legitimate responses to the task of compiling a multi‐donor, project‐level database. However, they will lead project fragmentation to be overstated to some degree in our work (other than regional comparisons, which are based on OECD data). Overstatement will not be major and we have no reason to believe it affects our substantive findings. Nevertheless, this will be an area for improvement in subsequent work.

## 4 RESULTS

In this section we report on our results, first looking at the question of whether aid to the countries of the Pacific is more volatile and fragmented than is the case with aid to other regions. We then look at countries within the Pacific, starting by studying trends over time in aid fragmentation, then reporting on aid fragmentation and volatility in individual countries. In the final section of our analysis, we look at aid donors and identify the major donors most guilty of giving fragmented and volatile aid. We then look at the question of whether non‐traditional donors such as China are more prone to giving volatile and fragmented aid.

### 4.1 The Pacific compared

Table 1 is based on OECD data. It looks at the level of aid fragmentation and volatility in the median aid recipient country in each of the world's main aid recipient regions. Two fragmentation measures are used: the HHI of donor fragmentation, and the number of projects per million people as a measure of project fragmentation. Because the data come from the OECD, key non‐traditional aid donors such as China are not included in the calculations. 4 However, as a starting assumption, it seems reasonable to anticipate that, as a group, those donors not in the OECD data will impact on each region in a broadly similar manner. In addition to measures, ranks are provided for each region. In all instances, the rank of 1 pertains to the worst region—the region where volatility or fragmentation is worst by the measure used.

**TABLE 1:** Regional measures of fragmentation and volatility

Region	Volatility		Donor fragmentation (HHI)		Project fragmentation	
	Median dyad	Rank	Median recipient	Rank	Median recipient	Rank
Africa	50.8	4	0.15	1	94.6	4
America	55.3	2	0.24	3	126.9	3
Asia	50.2	5	0.17	2	42.4	5
Europe	54.8	3	0.24	3	237.6	2
Pacific	68.7	1	0.44	5	2559.9	1

Abbreviation: HHI, Herfindahl–Hirschman Index.

The measure of fragmentation reported in Table 1 is fragmentation in the median recipient in each region. This is the case for both donor and project fragmentation. As discussed in the methodology, our primary interest in this article is in volatility at the level of the donor‐recipient dyad. As a result, in this table we focus on the median donor‐recipient dyad in each region, rather than volatility of total flows to a region or some similar measure. 5

As can be seen in Table 1, in terms of donor fragmentation, the Pacific is actually the region that suffers the least fragmentation. Project fragmentation on the other hand is much worse in the Pacific than elsewhere. Once their size is taken into account, the countries of the Pacific see their aid fragmented across many more projects than do aid recipients in other regions. Aid volatility is also worse in the Pacific than in other regions.

### 4.2 Fragmentation and volatility within the Pacific

**FIGURE 1:**
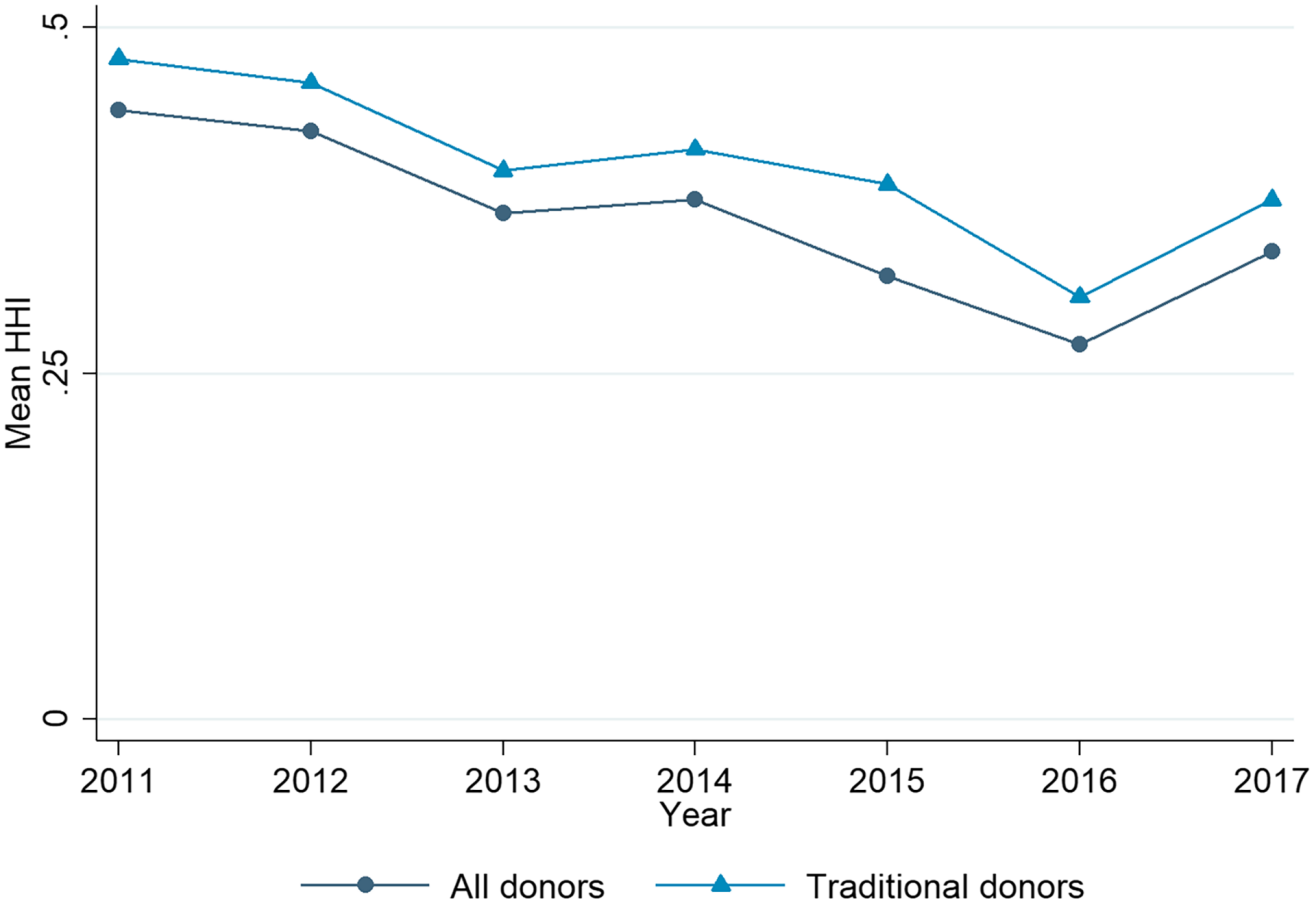
HHI trends in the Pacific, 2011–2017. Abbreviation: HHI, Herfindahl–Hirschman Index

Because volatility is calculated over time, and because the Lowy aid data only span 7 years, meaning the time series is too short for volatility over time to be analysed for shorter periods, we cannot assess trends in volatility over time using the Lowy data. However, we can look at trends in aid fragmentation. Figure 1 shows trends in fragmentation as experienced by Pacific countries, measured with the HHI. The figure shows the mean HHI averaged across Pacific countries for each year from 2011 to 2017.

The lower of the two lines on the figure, which is based on data from all donors, reveals a clear trend of greater fragmentation from 2012 to 2016 (recall that a lower HHI reflects greater fragmentation). This trend only reversed slightly between 2016 and 2017. A similar trend emerges when the median recipient is used instead of the mean. It might be tempting to ascribe this rise in fragmentation to the rise of non‐traditional donors in the Pacific. However, the upper line on the chart is plotted based on fragmentation from traditional donors only. As can be seen, excluding non‐traditional donors does lead to somewhat better fragmentation levels overall. Yet the trend of increasing fragmentation is still present when non‐traditional donors are excluded.

**FIGURE 2:**
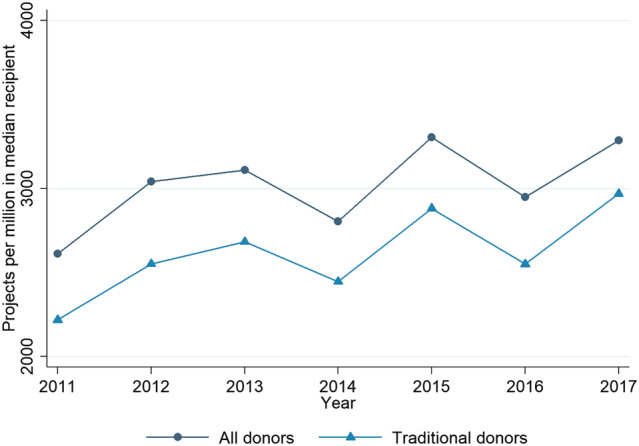
Projects per million people in the median Pacific country

Figure 2 charts project‐level fragmentation, measured as projects per million people in the median Pacific country. (The median country is chosen because some small countries have very high projects per million, leaving the median a more representative average. However, changing this measure to the mean country does not impact dramatically on the observed trend.)

As can be seen the number of projects, normalised by the number of people, fluctuated year on year, but the overall trend is of increase. Project fragmentation is becoming worse in the Pacific. Once again, as can be seen in the figure, when we replicated this exercise excluding non‐traditional donors, we found that the level of project fragmentation fell to some extent. However, the upwards trend was still clear in the data. The rise in project fragmentation in the Pacific is not being driven by non‐traditional donors.

**FIGURE 3:**
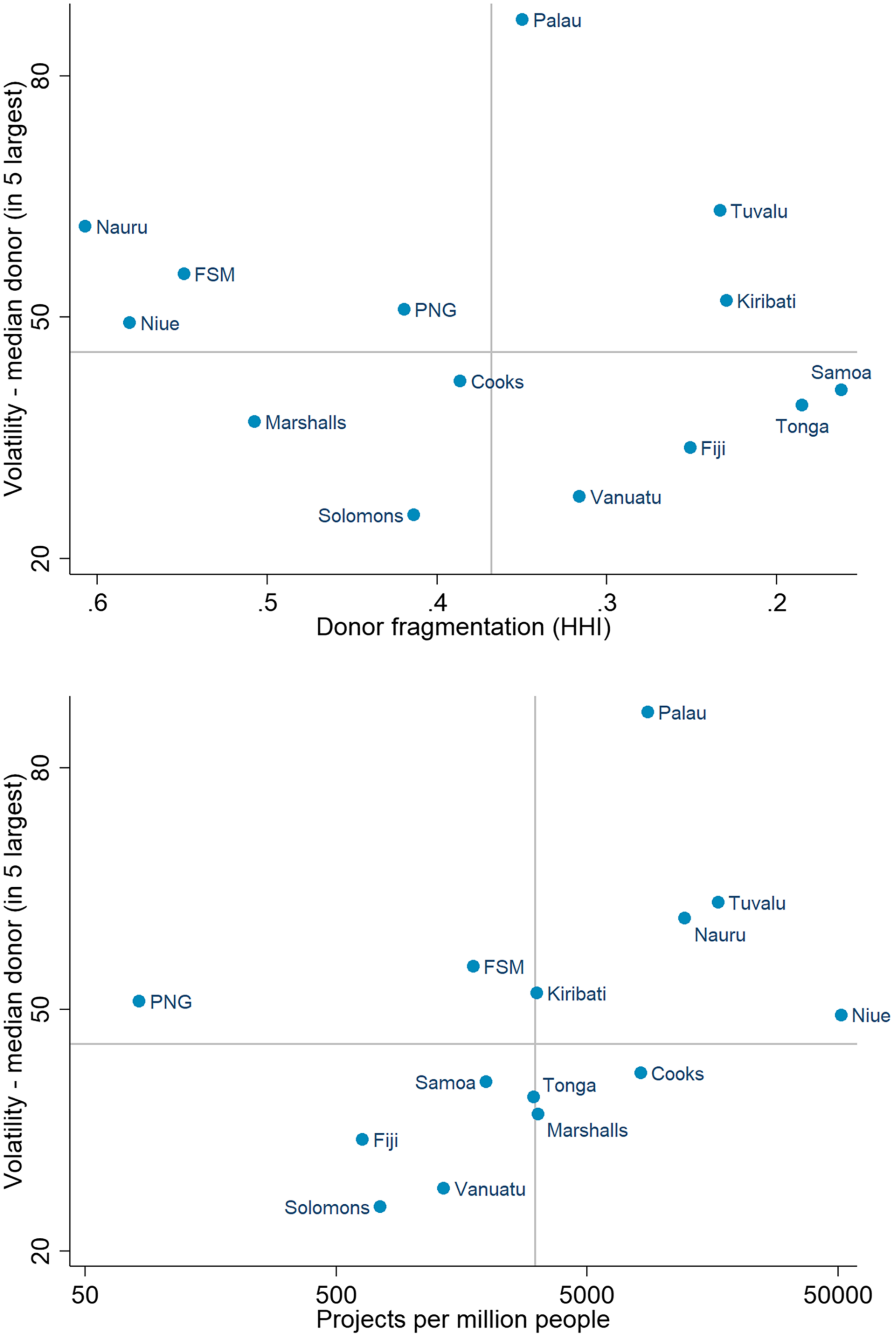
Volatility and fragmentation in the Pacific, 2011–2017. Abbreviations: FSM, Federated States of Micronesia; PNG, Papua New Guinea

Within the regional patterns identified in the previous sections, there is considerable variation. Some Pacific countries receive aid that is much more fragmentated and or volatile than others. Figure 3 shows two quadrant charts. Volatility is shown on the y‐axis of both charts. Because Figure 3 captures volatility measured as something that negatively impacts on aid recipients, and because volatility among minor donors will have much less of an impact than volatility among major donors, the measure shown is the volatility of the median donor (in terms of volatility) selected from the five largest donors (in terms of aid volume) to the recipient in question. HHI fragmentation is shown on the x‐axis of the first chart. Project fragmentation is shown on the x‐axis of the second chart. Each point on the chart is a Pacific country. The x‐axis is reversed in the first chart so that the chart reads intuitively, with countries that receive more fragmented aid to the right of the chart. The x‐axis scale in the second chart is logarithmic so as to span the huge range in project fragmentation contained within the chart. The x‐axis is also reversed in the second chart. Countries to the right of the chart suffer worse project fragmentation. On both axes in each chart, the gridlines are placed close to the median. Non‐traditional donors are included in the calculations in both charts. Humanitarian emergency aid is excluded.

As the top chart in Figure 3 makes clear, there is no obvious relationship between fragmentation across donors as measured by the HHI and aid volatility. Aid from the median donor to Nauru is volatile despite the country benefitting from little fragmentation across donors. Aid from the median donor to Vanuatu, on the other hand, is quite stable despite relatively high donor fragmentation.

While there is no obvious relationship between volatility and donor fragmentation, the top chart in Figure 3 does afford an explanation as to why some countries have less donor fragmentation than others. All of those countries with low fragmentation either have special status with particular donor countries (Niue and Cook Islands are realm states of New Zealand; the Federated States of Micronesia and Marshall Islands have a similar relationship with the United States), or Australia—the largest donor in the region—has a specific interest in the country in question. (Papua New Guinea is Australia's closest neighbour and a former colony. Australia led a regional policing mission to Solomon Islands. And Nauru has served as a major recipient of Australian asylum seekers.)

Similarly, the bottom chart in Figure 3 hints at a partial explanation for project fragmentation: most of the countries that suffer project fragmentation the worst have low populations, while those that suffer it the least are more populous. (We tested for this formally with a bivariate regression and found a clear relationship, with a *p*‐value of <0.01.) Although the evidence was not as clear cut when we tested, we also found suggestive evidence of a relationship between population and volatility—everything else being equal, volatility tends to be worse in smaller countries.

The pattern in the bottom chart in Figure 3 also hints at a relationship between project fragmentation and volatility. Formal testing provides some evidence to confirm this. The relationship between volatility and project fragmentation is close to being statistically significant at the 10% level. When Papua New Guinea, a clear outlier, is excluded the relationship becomes statistically significant at *p* < 0.05. Similarly, when the regressions are run controlling for population, the relationship between project fragmentation and volatility is statistically significant at *p* < 0.05. On average, aid is more volatile in those Pacific countries where project fragmentation is high.

One final point emerges from the two charts in Figure 3 viewed together. This is that there is no clear relationship between donor fragmentation as measured by the HHI and project fragmentation. In Pacific countries, greater fragmentation across donors does not mean greater project fragmentation as measured in this study. (Once again, we formally tested for this relationship regressing the two variables and found no evidence of a relationship.)

### 4.3 Fragmentation among donors in the Pacific

On the basis of the Lowy dataset, over the years 2011 to 2017, a total of at least 57 donors gave aid to the Pacific. Of these 57 donors, 52 spent money on development projects (i.e., projects that were not primarily responses to humanitarian emergencies). The range in size within this group is vast. Australia had something in the vicinity of 15,000 discrete projects over the period, and spent over US$6.5 billion in the region. Hungry, the smallest donor, contributed to one project and spent US$434. Reflecting such diversity, the extent to which donors fragment their aid across recipients and across projects varies considerably. Aid volatility is similarly varied.

**FIGURE 4:**
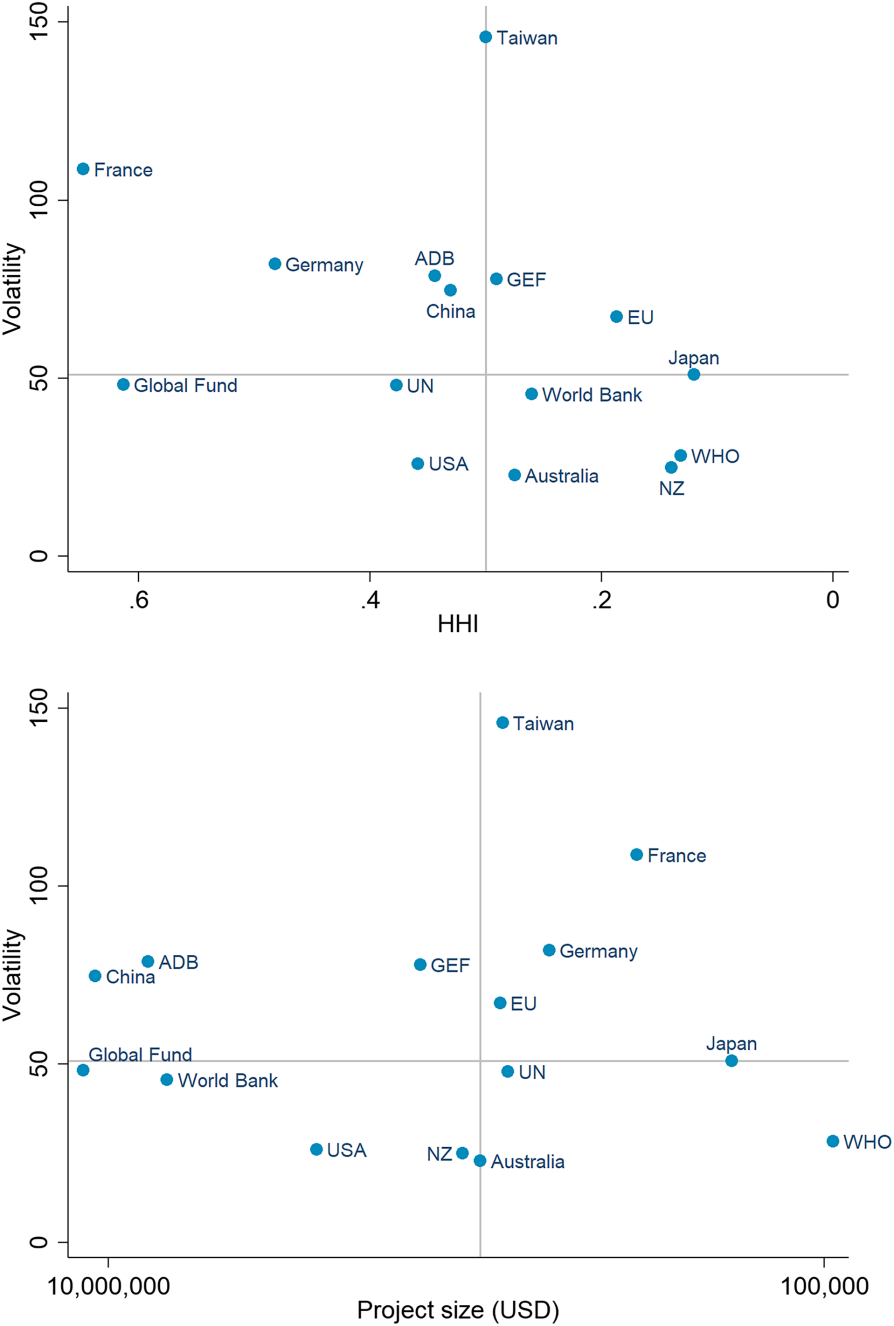
Volatility and fragmentation among donors, 2011–2017. Abbreviations: ADB, Asian Development Bank; EU, European Union; GEF, Global Environment Facility; NZ, New Zealand; UN, United Nations; USA, United States of America; WHO, World Health Organization

Figure 4 is similar to Figure 3, except that the points on the charts in the figure are donors. For legibility's sake, only the largest 15 donors to the Pacific (in terms of total aid over the period 2011 to 2017) are included in the figure. The impact of these larger donors on the region is much greater than that of their smaller counterparts—together they gave nearly 40 times more aid than the smaller donors in the Lowy dataset. For this reason, focusing on larger donors in the figure is reasonable. A full table of all measures for all donors can be produced using the data in the online dataset.

The volatility measure in Figure 4 reflects how volatile aid is from each donor in the median recipient country (in terms of the donor's volatility). The HHI measure in the top chart in the figure captures the extent to which each donor fragments their aid across the countries of the Pacific. The project fragmentation measure in the second chart in the figure is the mean project size for each donor (averaged across all their projects in the region). As discussed in the methodology section, this measure of project fragmentation is designed to account for the fact that it is reasonable enough for donors that give the region a greater volume of aid to have more projects. Mean project size accounts for this. 6

Among the donors charted, Taiwan stands out for giving particularly volatile aid, while the United States, New Zealand, Australia and the World Health Organization (WHO) are the most stable donors. New Zealand, Japan and the WHO all fare poorly on the HHI measure. Japan and the WHO also fare poorly on the project fragmentation measure. New Zealand does much better on this measure. China gives fairly volatile aid, but performs better than average on the HHI measure, and much better than average on the project size measure—alongside the development banks and the Global Fund, it forms part of a small group of donors whose projects are very large on average.

If the outlier of Taiwan is ignored, a weak relationship exists between volatility and HHI—donors that have worse HHI scores tend to give less volatile aid. However, it should be emphasised this relationship is weak, and ceases to exist if the sample is expanded beyond the 15 largest donors. There is no relationship between volatility and average project size. Nor is there a relationship between HHI fragmentation and project size fragmentation.

Table 2 summarises three regression models run to examine the relationships between other key variables and the measures of donor fragmentation and volatility used in this study. The regressions are run using all donors, not just the largest 15. The results in the table show that larger donors (with size measured as the natural log of total aid given) tend, on average, to fragment their aid more across Pacific recipient countries (as measured by HHI). At the same time though, larger donors also tend to have larger projects—therefore performing better on the measure of project fragmentation.

**TABLE 2:** Correlates of fragmentation and volatility (all donors)

	HHI	Project size (mean)	Volatility
New donor	−0.07 (0.41)	466,678 (0.61)	79.14*** (0.00)
Total aid (natural log)	−0.05*** (0.00)	341,956*** (0.00)	−3.68 (0.20)
Constant	1.42*** (0.00)	−4,118,917** (0.02)	135.56*** (0.01)
Observations	52	52	37
R‐squared	0.41	0.20	0.25

Abbreviation: HHI, Herfindahl–Hirschman Index.

*p*‐values in parentheses

* *p* < 0.1.

** *p* < 0.05.

*** *p* < 0.01.

The other variable tested in the regression models is whether a donor is a ‘traditional donor’ (defined as countries that are members of the OECD DAC, as well as multilateral organisations that report to the OECD such as the World Bank and Asian Development Bank) or whether a donor is a ‘non‐traditional’ donor such as Taiwan, India or China. As can be seen in the first two models, there is no evidence that non‐traditional donors give aid that is more fragmented, on average. However, the third model provides clear evidence that non‐traditional donors are currently giving more volatile aid. 7

## 5 DISCUSSION

The Pacific is not only aid dependent, but as a region it also suffers worse aid volatility and project fragmentation than other aid recipient regions do. Pacific countries tend to suffer less donor fragmentation than countries in other aid recipient regions. Even here, there is little cause for complacency. Limited donor fragmentation likely stems from the fact the Pacific was for a long time of limited geostrategic interest to donors. Raised concerns about China's presence in the region may well mean this is about to change (Dornan, 2018). And as we have shown in this article, donor fragmentation is increasing in the Pacific. At the same time, fragmentation across projects is also increasing.

Donors and other aid actors concerned with effective aid need to pay clear attention to aid fragmentation and volatility in the Pacific.

This article, and the data that accompany it, are intended as a starting point in paying more attention to volatility and fragmentation in the region. In addition to contextualising the extent of fragmentation and volatility in the Pacific by comparing the Pacific to other regions, and in addition to establishing overall trends of worsening fragmentation, we have found that the problems we have identified in the region cannot solely be attributed to non‐traditional donors. We found fragmentation to be increasing even when we restricted our analysis to traditional OECD DAC country donors and multilaterals. We also found that project and country fragmentation is not higher among non‐traditional donors than it is among traditional donors. Only aid volatility is a worse issue among non‐traditional donors.

Studying recipients, it appears that countries that have special ties—whether these are formal, historical, or in some instances strategic—to key donors suffer less donor fragmentation. It is clearly the case that smaller Pacific countries suffer worse project fragmentation. In the smallest Pacific countries, some additional project fragmentation may be unavoidable: the need for different projects in different sectors with different objectives means that aid cannot be given in singular lump sums to countries such as Niue. At the same time, however, the truly small Pacific states are the countries that can least afford project fragmentation and the strains it places on human resources and recipient government engagement. Additional cause for concern regarding project fragmentation, particularly in the smallest recipient countries, comes from the potential relationship between project fragmentation and volatility. Although we have not definitively proved that greater project fragmentation causes greater aid volatility, the relationship we identified between the two phenomena and the fact that both are most acute in the smallest Pacific countries provides additional cause for concern regarding project fragmentation.

One possible means that aid fragmentation and volatility could be tackled as issues in the Pacific is simply through better coordination between donors and better donor forward planning. Such solutions may be able to mitigate the negative effects of fragmentation and volatility without necessitating project consolidation, fewer donors, or more stable aid flows. Coordination and planning will certainly be important in working to address the issues we have identified in this article. As Dornan and Pryke (2017) show, however, coordination and forward planning have not been donor strongpoints in the Pacific. Unless coordination and planning improve substantially, these means will not be enough to offset the problems of fragmentation and volatility.

Above and beyond existing attempts at planning and coordination we recommend the following.

First and foremost, we urge traditional donors to respond in a considered manner to their rising concerns about the presence of China in the Pacific. At the time of writing, a queue of traditional donors, including Australia, New Zealand, the United States and some European countries, are moving to devote more aid attention to the Pacific. A surge of renewed interest in the region from OECD DAC donors seeking to stave off perceived Chinese influence will not help with fragmentation. Similarly, an overly reactive stance to China's presence may pre‐empt possible coordination between traditional and non‐traditional donors in the future—coordination that will be needed if fragmented aid is not to reduce aid effectiveness in the Pacific. Increased aid to the Pacific has the potential to be of real value. However, it should be driven by development needs, not geostrategic impulses. Donors also need to strive to coordinate more effectively than has previously been the case if they do not want increased fragmentation to undermine aid's development benefits.

Second, donors need to carefully reconsider their approach to aid projects in the Pacific's smallest countries, striving to maximise predictability and minimise project proliferation. We have noted that some project fragmentation is probably inevitable in the smallest Pacific countries; however, fragmentation should be no more than is absolutely necessary. Trust funds and similar tools should be adopted where feasible to minimise fragmentation in the smallest Pacific countries.

Third, more study, possibly supported by donors or regional institutions, is clearly warranted. Further quantitative work would be useful—both to add to the measures we have drawn upon here, and also to ensure that the issues of fragmentation and volatility continue to have a high profile. More studies of the sort conducted by Iulai (2014) that carefully identify the actual impacts of fragmentation and volatility in Pacific aid recipients also have the potential to add much value. Qualitative studies within donor organisations would also be very helpful: there is a lot to learn about just why aid becomes fragmented and volatile, and what processes within aid donors can help drive improvements.

Regardless of the specifics, as we have shown here, aid fragmentation and volatility need concerted, continued attention in the Pacific. We have demonstrated a problem exists, both in terms of levels and trends. And we have developed a new dataset which we hope will be used as aid actors work to ensure that fragmentation and volatility do not unduly diminish aid's potential to assist the countries of the Pacific.

## Data Availability

The data that support the findings of this study are openly available in figshare at http://doi.org/10.6084/m9.figshare.11678022, reference number 11678022.

## Footnotes

^1^ Aid dependency here is calculated as aid/Gross National Income. Data are from 2017, the most recent available year at the time of writing. Data come from the OECD: https://stats.oecd.org/Index.aspx?datasetcode=TABLE2A.

^2^ For any individual donor the HHI is calculated as follows: $\mathrm{HD}=\sum_{c=1}^{N}r_{c}^{2}$, where *HD* is the HHI for the donor, *N* is the number of aid recipient countries in the Pacific the donor gives aid to and *r* is the share of the donor's aid to the Pacific given to country *c*. For a recipient the equivalent formula is used except the share of funding from each individual donor is squared then summed.

^3^ For a helpful explanation of how this approach is put into practice, see https://stats.idre.ucla.edu/other/mult-pkg/faq/general/faq-what-is-the-coefficient-of-variation/.

^4^ Other non‐DAC donors such as Taiwan do report to the OECD; however, where available their data are included in calculations.

^5^ As an alternative approach to regional comparisons we also investigated volatility of total aid flows to recipient countries. When we did this the Pacific was the median region for volatility (in its median recipient country). Aid to the Americas was more volatile and aid to Africa was slightly more volatile.

^6^ Because a small number of projects are multi‐country and some projects involve spending across multiple years, average project size was calculated at the project level, for individual projects, aggregating spending across recipient countries and years. The mean was used. We investigated using median project size. Measures of mean and median project size were very strongly correlated across donors. As a result, both measures generate broadly similar results.

^7^ A number of non‐traditional donors did not give aid for three or more years to any Pacific countries, and so do not contribute to volatility data (Thailand is one example). However, the four most important non‐traditional donors (Taiwan, China, India and the United Arab Emirates) were included in the analysis.

## References

[app5321-bib-0001] Birdsall, N. (2005). Seven deadly sins: Reflections on donor failings. Center for Economic and Policy Research Briefing Paper No 50, 1–37. https://www.cgdev.org/publication/seven-deadly-sins-reflections-donor-failings-working-paper-number-50

[app5321-bib-0002] Bulíř, A. , & Hamann, A. J. (2008). Volatility of development aid: From the frying pan into the fire? World Development, 36(10), 2048–2066. 10.1016/j.worlddev.2007.02.019

[app5321-bib-0003] Clarke, M. , Fry, T. , & Mihajilo, S. (2008). The volatility of aid to small island states. Pacific Economic Bulletin, 23(2), 179–202. http://devpolicy.org/PEB/2019/07/03/the-volatility-of-aid-to-small-island-states/

[app5321-bib-0004] Dayant, A. , & Pryke, J. (2019). Pacific aid map. Lowy Institute. https://pacificaidmap.lowyinstitute.org/

[app5321-bib-0005] Dornan, M. (2018, June 25). Australia's relationships with its Pacific Island neighbours should not be about China. *Devpolicy Blog*. https://devpolicy.org/australias-relationships-with-pacific-should-not-be-about-china-20180625/

[app5321-bib-0006] Dornan, M. , & Pryke, J. (2017). Foreign aid to the Pacific: Trends and developments in the twenty‐first century. Asia & the Pacific Policy Studies, 4(3), 386–404. 10.1002/app5.185

[app5321-bib-0007] Easterly, W. , & Pfutze, T. (2008). Where does the money go? Best and worst practices in foreign aid. The Journal of Economic Perspectives, 22(2), 29–52. 10.1257/jep.22.2.29

[app5321-bib-0008] Gehring, K. , Michaelowa, K. , Dreher, A. , & Spörri, F. (2017). Aid fragmentation and effectiveness: What do we really know? World Development, 99, 320–334. 10.1016/j.worlddev.2017.05.019

[app5321-bib-0009] Hudson, J. (2015). Consequences of aid volatility for macroeconomic management and aid effectiveness. World Development, 69, 62–74. 10.1016/j.worlddev.2013.12.010

[app5321-bib-0010] Hudson, J. , & Mosley, P. (2008). Aid volatility, policy and development. World Development, 36(10), 2082–2102. 10.1016/j.worlddev.2007.02.018

[app5321-bib-0011] Iulai, L. (2014). Aid volatility: Is it a problem in Tuvalu? Asia & the Pacific Policy Studies, 1(2), 379–394. 10.1002/app5.30

[app5321-bib-0012] Kimura, H. , Mori, Y. , & Sawada, Y. (2012). Aid proliferation and economic growth: A cross‐country analysis. World Development, 40(1), 1–10. 10.1016/j.worlddev.2011.05.010

[app5321-bib-0013] Knack, S. (2008). Donor fragmentation and aid effectiveness. A brief from the World Development Group, World Bank. http://documents1.worldbank.org/curated/en/612461468057845806/pdf/539110BRI0knac10Box345633B01PUBLIC1.pdf

[app5321-bib-0014] Knack, S. , & Rahman, A. (2007). Donor fragmentation and bureaucratic quality in aid recipients. Journal of Development Economics, 83(1), 176–197. 10.1016/j.jdeveco.2006.02.002

[app5321-bib-0016] Organization for Economic Co‐operation and Development . (2008). *The Paris Declaration on Aid Effectiveness and Accra Agenda for Action*. OECD. https://www.oecd.org/dac/effectiveness/34428351.pdf

[app5321-bib-0015] Organization for Economic Co‐operation and Development . (2009). Development co‐operation report 2009. OECD Publishing. 10.1787/dcr-2009-en

